# Impact of Side Chain Polarity on Non-Stoichiometric Nano-Hydroxyapatite Surface Functionalization with Amino Acids

**DOI:** 10.1038/s41598-018-31058-5

**Published:** 2018-08-23

**Authors:** Patricia Comeau, Thomas Willett

**Affiliations:** Composite Biomaterial Systems Laboratory, Department of Systems Design Engineering, University of Waterloo, Waterloo, ON N2L 3G1 Canada

## Abstract

In this study the affinity of three amino acids for the surface of non-stoichiometric hydroxyapatite nanoparticles (ns-nHA) was investigated under different reaction conditions. The amino acids investigated were chosen based on their differences in side chain polarity and potential impact on this surface affinity. While calcium pre-saturation of the calcium-deficient ns-nHA was not found to improve attachment of any of the amino acids studied, the polarity and fraction of ionized functional side groups was found to have a significant impact on this attachment. Overall, amino acid attachment to ns-nHA was not solely reliant on carboxyl groups. In fact, it seems that amine groups also notably interacted with the negative ns-nHA surface and increased the degree of surface binding achieved. As a result, glycine and lysine had greater attachment to ns-nHA than aspartic acid under the reaction conditions studied. Lastly, our results suggest that a layer of each amino acid forms at the surface of ns-nHA, with aspartic acid attachment the most stable and its surface coverage the least of the three amino acids studied.

## Introduction

The mechanical properties, biocompatibility, and osteoconductive properties of hydroxyapatite (HA) alone has led to extensive research towards the use of this material in biomedical and industrial applications such as in orthopaedics^1,2^. Non-stoichiometric carbonated hydroxyapatite in particular is garnering increased attention for use in biomedical applications owing to its greater chemical similarity to the mineral phase of bone (compared to stoichiometric hydroxyapatite)^3^. By taking advantage of the adsorption properties of nHA, the surface chemistry of these nanoparticles may be tuned by using a functionalization approach with water-soluble biomolecules. In this study, we report on the binding of different amino acids to a non-stoichiometric carbonated nHA (ns-nHA) surface using different reaction conditions.

Amino acids have previously been proposed as ideal candidates for such an approach as a result of their amphoteric nature and ability to interact with HA surfaces, as well as their low cost and intrinsic biocompatibility^1,4^. Prior studies have largely focused on the effect of amino acids on HA crystallization, with negatively charged amino acids (e.g. aspartic acid) commonly being shown to control HA mineralization in bone most effectively^5–9^. However, there are contradictory results reported in the literature on the interaction of amino acids with HA, such that a comprehensive understanding of the mechanisms involved remains largely elusive. For example, Jack *et al*.^2^ reported that a positively charged amino acid had the strongest affinity for the HA surface, while in a series of papers Koutsopoulos and Dalas^5–8,10^ reported that the largest affinity was with a negatively charged amino acid (specifically aspartic acid). More recent studies have begun to emphasize the impact of side chain functionalities and how interactions at the surface of stoichiometric HA may be maximized as a result^9,11,12^. For example, in a simulated modelling study, Rimola *et al*.^11^ reported that, as a result of side chain interactions with HA, Lysine and other investigated polar amino acids had a greater surface stability than non-polar glycine. How such findings may apply to ns-nHA remains inconclusive and more investigation is necessary. To address these inconsistencies, this study was conducted to further our understanding of amino acid affinity with the ns-nHA surface and to further realize any side group dependence on this affinity.

Unfortunately, functionalizing nHA utilizing the more traditional crystallization approach has been found to interfere with ion attachment on certain crystal faces and to create additional defects or inconsistencies in the HA crystal structure, as well as lead to reduced crystallinity^13–16^. Intrinsic factors such as defect density are known to reduce material strength^17^, and could compromise the mechanical performance of not only the mineral phase (e.g. nHA), but ultimately any future composite. Owing to our interest in pursuing composite development with the functionalized ns-nHA, this study sought the development of a more direct approach to functionalizing the surface of ns-nHA.

Hydrogen bonding, as well as electrostatic and van der Waals interactions between amino acids and nHA govern the adsorption of amino acids onto the nHA surface. Analysis of the electrostatic potential at the HA surface has previously revealed a highly positive potential on top of calcium ions and deep negative zones in the proximity of oxygen atoms of the PO_4_ groups^11^. As a result, the strength of interactions is primarily dependent on such surface properties of nHA, but may also be influenced by experimental conditions including pH of the medium and type of amino acid^18^. For example, as a result of the different side chain groups, each amino acid possesses a different isoelectric point (pI) and, hence, unique net surface charge^4^. In this study, we investigated the affinity of three different amino acids for the surface of ns-nHA under different experimental pH conditions and following pre-saturation of ns-nHA with calcium. We hypothesized that should the carboxyl interaction with ns-nHA surface calcium ions be the dominant attachment mechanism, the more carboxyl groups provided by the amino acid and the lower the reaction pH, the greater the coverage of ns-nHA with the amino acids. Similarly, by including a ns-nHA pre-saturation step with divalent calcium ions, we also hypothesized that functionalization of ns-nHA would be enhanced with dependence on initial reaction pH. The pre-saturation of the as-received ns-nHA with calcium will also allow for the observation of an assumed stoichiometry impact of the nHA surface on amino acid adsorption.

## Results

### Confirming the Particle Size and Ca/P ratio of As-received and Unmodified ns-nHA

XRD analysis of our starting, dry powders reported a crystallinity of 66% following the approach by Del Valle *et al*.^19^, while BET (Brunauer, Emmett and Teller) analysis revealed a surface area of 89.91 m^2^/g. In addition, scanning electron microscopy (SEM) confirmed that the particles were of rod-like shape (see Supplementary Information, Fig. S1), while from SEM with energy dispersive x-ray (SEM-EDX) analysis the molar ratio of Ca/P was found to be 1.52 and the ns-nHA was confirmed as being carbonated (see Supplementary Information, Fig. S2). SEM analysis also revealed that the ns-nHA particles were ~120 nm in length and 20–30 nm in width on average.

### Zeta Potential of ns-nHA becomes Less Negative upon Amino Acid Functionalization

**Figure 1:**
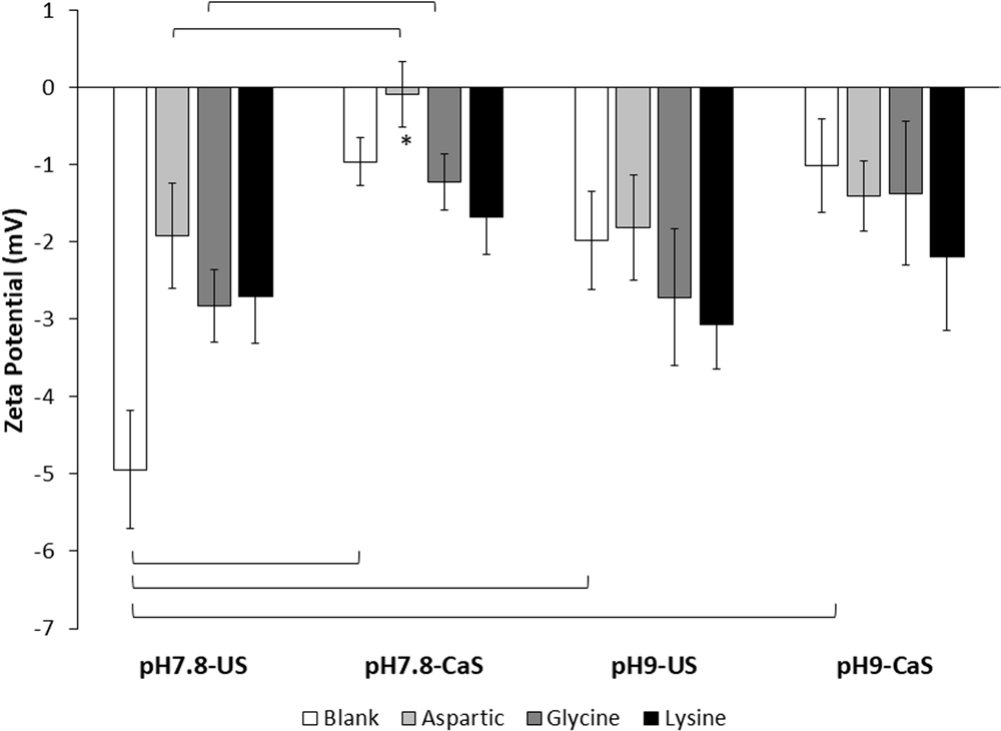
Zeta potential analysis of functionalized ns-nHA. Pre-conditioning of blank ns-nHA and addition of any of the amino acids studied notably increased the zeta potential (i.e. made it less negative). Of particular note is the near-neutral zeta potential of aspartic acid functionalized ns-nHA prepared under calcium-saturated and pH 7.8 conditions (indicated by *). This balanced charge likely results from a more balanced binding of both carboxyl and amine groups to the ns-nHA surface (compared to the other amino acids). Data reported as average ± one standard deviation (n = 5). ns-nHA unsaturated with calcium is given as US, while that pre-saturated with calcium is given as CaS. Horizontal bars indicate statistically detectable difference (p < 0.05).

Functionalization of the ns-nHA surface using amino acids resulted in changes to the local surface charge due to the presence of unbound COO^−^ and NH_2_-H^+^ groups from the amino acids, as well as differences in the amounts of vacant grafting sites on the ns-nHA surface (Fig. 1).

A three factor general linear model indicated that the interaction between reaction pH and both calcium saturation and amino acid type (both p < 0.0001), as well as between calcium saturation and amino acid type (p = 0.001) was statistically detectable for zeta potential. In addition, the pre-saturation of ns-nHA with calcium and amino acid type were each independently found to impact zeta potential (both p < 0.0001). At stable reaction conditions (pH 7.8 and no pre-saturation with calcium), the addition of amino acids to the ns-nHA surface notably increased the zeta potential value (less negative) compared to the blank (i.e. unfunctionalized) ns-nHA. Furthermore, under calcium saturated and pH 7.8 conditions, the ns-nHA surface functionalized with aspartic acid had the greatest zeta potential of all sample groups. With a near-neutral surface charge, there is minimal electrostatic repulsion between the like-charged aspartic acid-functionalized particles. A more balanced aspartic acid layer on the ns-nHA surface, with carboxyl and amine groups of the amino acid both serving as binding agents, likely contributed to this reported zeta potential.

### ATR-FTIR COO^−^ Symmetric Stretch Peak Area Greatest for Aspartic Acid, while NH_2_-H^+^ Symmetric Stretch Peak Area Greatest for Lysine

**Figure 2:**
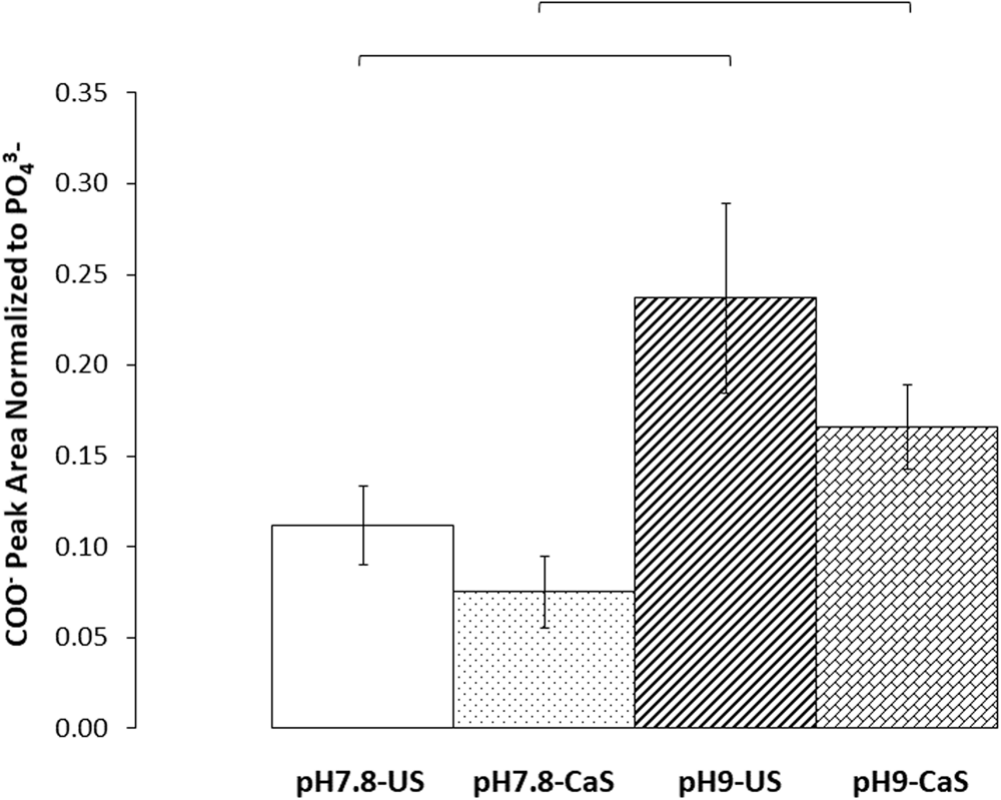
ATR-FTIR analysis of COO^−^ peak area normalized to PO_4_^3−^ peak area for blank ns-nHA. Increasing the reaction pH notably increased the ratio of COO^−^ symmetric stretch peak area to PO_4_^3−^ vibration peak area. The saturation of ns-nHA with calcium (CaS) did not result in a detectably different COO^−^ peak area compared to unsaturated (US). Data reported as average ± one standard deviation (n = 3). Horizontal bars indicate a statistically detectable difference (p < 0.05).

**Figure 3:**
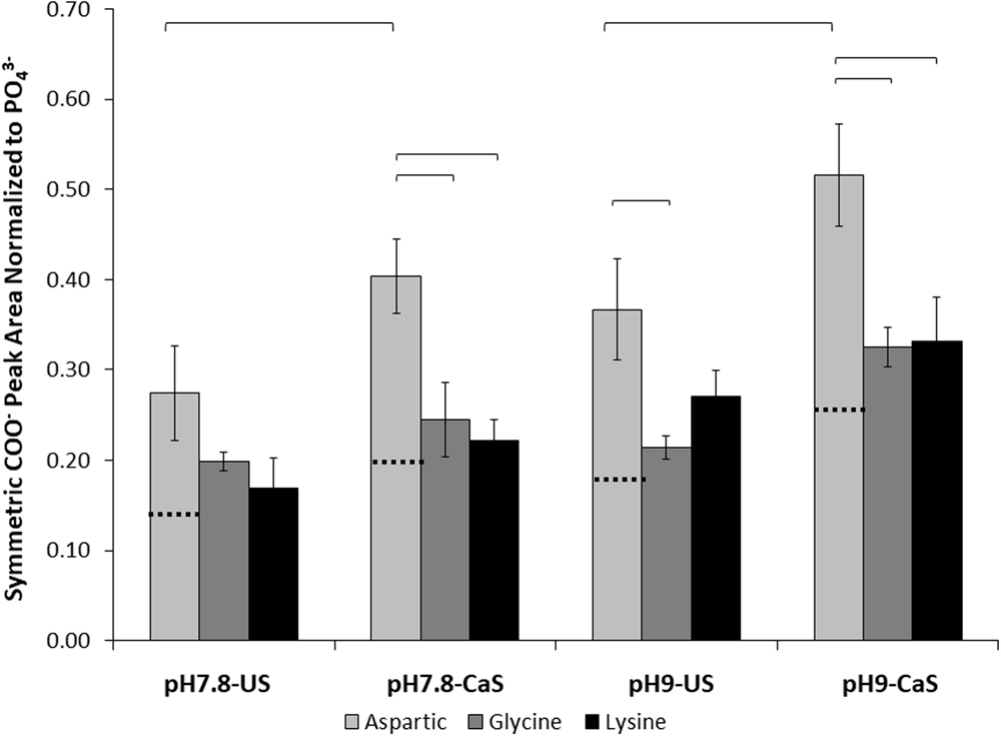
ATR-FTIR analysis of the amino acid functionalized ns-nHA COO^−^ peak area normalized to PO_4_^3−^ peak area and peak area of blank ns-nHA subtracted for each condition. Aspartic acid-functionalized ns-nHA was found to have the greatest symmetric COO^−^ peak area. However, there are two COO^−^ groups per aspartic acid molecule (glycine and lysine only have one COO^−^ group per molecule). As shown by the black horizontal dashed line on the aspartic acid column, the COO^−^ peak area analysis suggests that there is a comparable amount of aspartic acid attached to the ns-nHA surface as achieved with the other amino acids. Data reported as average ± one standard deviation (n = 3). ns-nHA unsaturated with calcium is given as US, while that pre-saturated with calcium is given as CaS. Horizontal bars indicate statistically detectable difference (p < 0.05).

**Figure 4:**
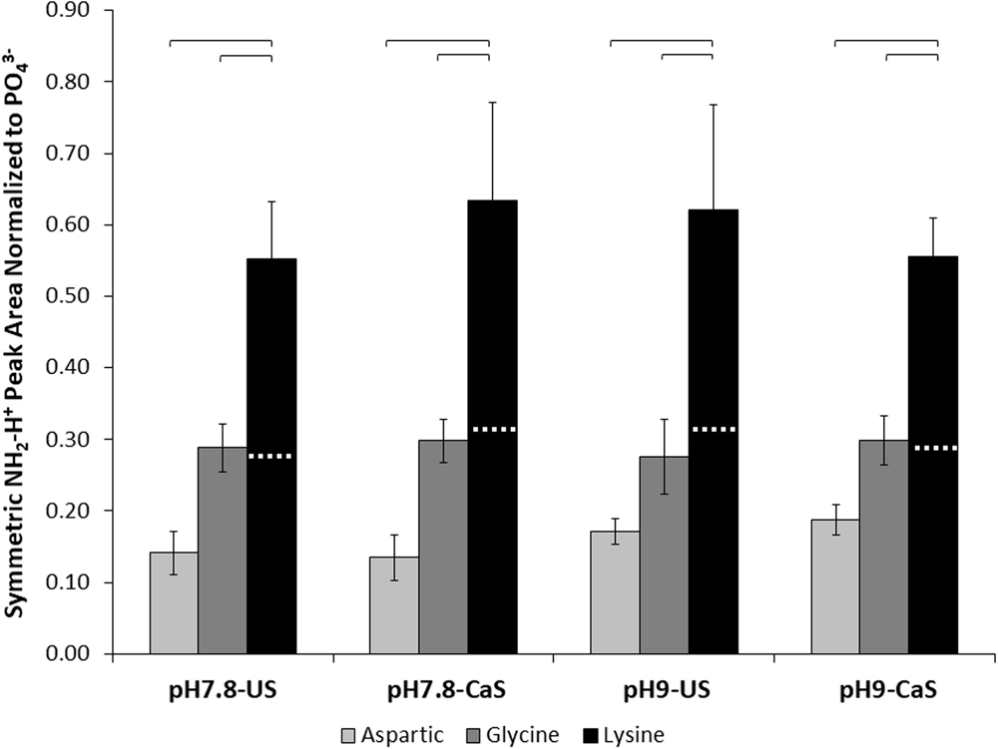
ATR-FTIR analysis of the amino acid functionalized ns-nHA NH_2_-H^+^ peak area normalized to PO_4_^3−^ peak area. Lysine-functionalized ns-nHA was found to have the greatest symmetric NH_2_-H^+^ peak area. However, there are two NH_2_-H^+^ groups per lysine molecule (aspartic acid and glycine only have one NH_2_-H^+^ group per molecule). As shown by the white horizontal dashed line on the lysine column, the NH_2_-H^+^ peak area analysis suggests that there is a comparable amount of lysine attached to the ns-nHA surface as glycine. Data reported as average ± one standard deviation (n = 3). ns-nHA unsaturated with calcium is given as US, while that pre-saturated with calcium is given as CaS. Horizontal bars indicate statistically detectable difference (p < 0.05).

ATR-FTIR analysis revealed further differences in the amino acid-dependent affinity to the ns-nHA surface (see Supplementary Information, Fig. S3, for a representative stacked plot). The results of COO^−^ peak area normalized to the 605 cm^−1^ PO_4_^3−^ vibration peak for the blank ns-nHA groups are given in Fig. 2. Meanwhile, the normalized COO^−^ and NH_2_-H^+^ symmetric stretch peak areas (with that of the blank ns-nHA subtracted, respectively) are given for the amino acid functionalized ns-nHA in Figs 3 and 4.

For the blank ns-nHA samples, reaction pH was found to have a statistically detectable impact on COO^−^ peak area (p = 0.0004) using a two-way ANOVA. A contributing factor to the increase in normalized peak area is the likely reduced amount of phosphate species present for the blank ns-nHA as a result of hydrolysis during the experiment. This also corresponds to the higher (less negative) zeta potential observed for pH 9 conditioned blank ns-nHA samples (Fig. 1) – having fewer phosphate species would reduce the amount of negative surface charge. Analysis of the pH change during reaction and characterization of the specific surface area (SSA) of the blank following functionalization supports the greater hydrolysis of ns-nHA at pH 9 (see Figs S4 and S5, respectively, in supplementary information). For example, the change in pH during reaction is greater for the blank ns-nHA at pH 9 than at pH 7.8.

A three-factor general linear model of the COO^−^ peak area data revealed that the interaction between amino acid type and pre-saturation of ns-nHA with calcium was statistically detectable (p = 0.043), while pH (p < 0.001), pre-saturation of ns-nHA with calcium (p < 0.0001), and amino acid type (p < 0.0001) each independently affected the normalized COO^−^ peak area. With twice as many carboxyl functional groups per aspartic acid molecule, it is congruent that the COO^−^ peak area for aspartic acid is almost twice as great as the other two amino acids (which only have one carboxyl functional group per molecule). In addition, for aspartic acid only, the pre-saturation of ns-nHA with calcium notably increased the COO^−^ peak area; however, upon normalization for the number of carboxyl groups per aspartic acid molecule this effect is largely eliminated. Meanwhile, this same statistical model indicated that only the amino acid type impacted the normalized NH_3_^+^ peak area (p < 0.0001). Here it is important to note that there are twice as many amine functional groups per amino acid molecule for lysine as glycine. As a result, with NH_2_-H^+^ peak areas almost twice as high for lysine as glycine, this then suggests that there was a similar amount of attachment to ns-nHA for these two amino acids (this is similarly supported by COO^−^ peak area).

### Amino Acid Attachment to ns-nHA Occurs via Both Carboxyl and Amine Functional groups

**Figure 5:**
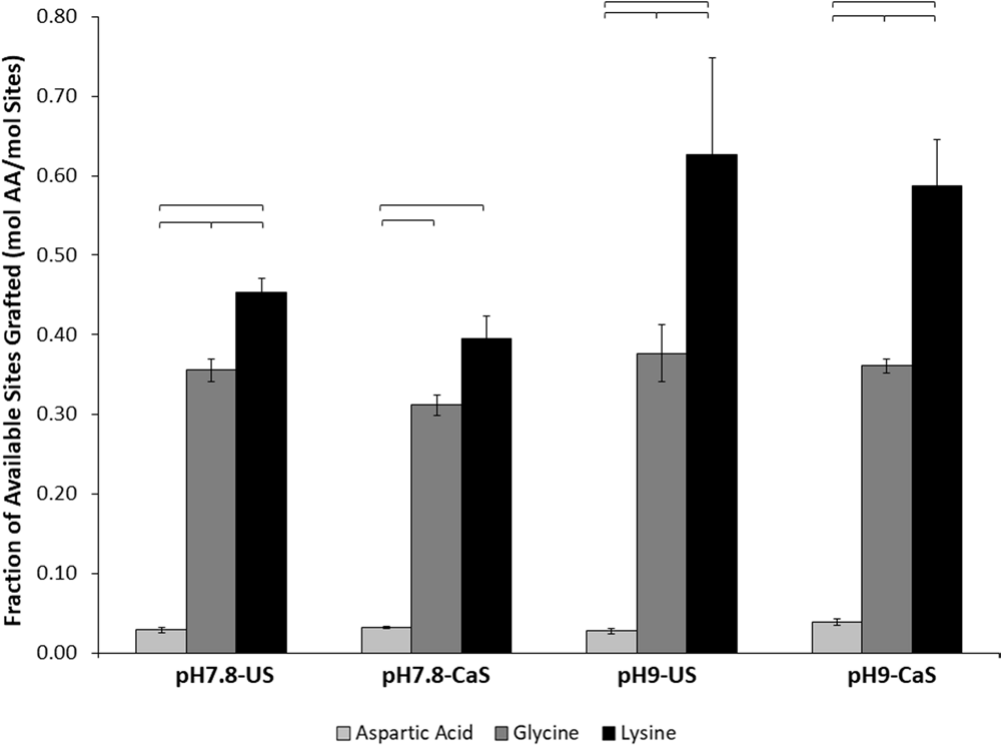
Molar ratio of total sites grafted on the ns-nHA surface using different amino acids (AA) following the ninhydrin protocol. Aspartic acid attached to fewer available graft sites on ns-nHA than glycine or lysine; this is likely indicative of the more balanced layer of aspartic acid on the ns-nHA surface. Data reported as average ± one standard deviation (n = 5). ns-nHA unsaturated with calcium is given as US, while that pre-saturated with calcium is given as CaS. Horizontal bars indicate statistically detectable difference (p < 0.05).

**Figure 6:**
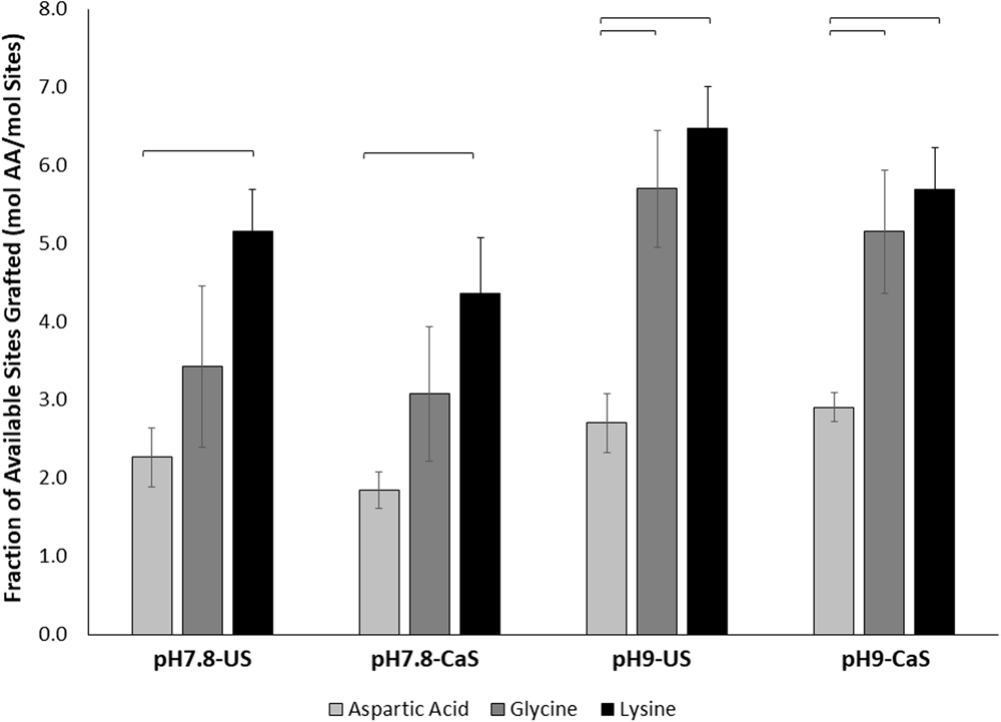
Molar ratio of total sites grafted on the ns-nHA surface using different amino acids (AA) following the fluoraldehyde protocol. Aspartic acid was again found to attach to fewer available graft sites on ns-nHA than the other two amino acids. Data reported as average ± one standard deviation (n = 5). ns-nHA unsaturated with calcium is given as US, while that pre-saturated with calcium is given as CaS. Horizontal bars indicate statistically detectable difference (p < 0.05).

The impact of the different reaction conditions on amino acid affinity for the ns-nHA surface was also quantified using a ninhydrin protocol^20^ which characterizes the amine groups free at the ns-nHA surface, and a fluoraldehyde method^21^ which involves the digestion of ns-nHA and subsequent accounting of all amine groups present with ns-nHA (attached or free at the ns-nHA surface). The fraction of moles of amino acid attached to moles of sites originally available for grafting on ns-nHA is shown in Fig. 5 for the ninhydrin protocol and Fig. 6 for the fluoraldehyde method.

A three-factor general linear model analysis of the ninhydrin protocol data revealed that the interaction of reaction pH and amino acid type is detectably different (p < 0.0001). Meanwhile, reaction pH (p < 0.0001), pre-saturation of ns-nHA with calcium (p = 0.036), and amino acid type (p < 0.0001) were each found to independently impact the fraction of sites grafted. As a result, only lysine sees a detectable increase in attachment to ns-nHA as pH increases. A similar model analysis of the fluoraldehyde assay data also indicated that the interaction of reaction pH and amino acid type was detectable (p = 0.004). Furthermore, and as with the ninhydrin protocol, reaction pH (p < 0.0001), pre-saturation of ns-nHA with calcium (p = 0.009), and amino acid type (p < 0.0001) were each found to independently impact the fraction of sites grafted as per the fluoraldehyde assay.

The trend in the ratio of moles of amino acid detected to sites originally available for grafting on the surface of ns-nHA is similar for the ninhydrin and fluoraldehyde protocols. It is important to recognize that the use of two different protocols to quantify amino acid attachment to nHA limits the ability to compare pre- and post-digestion assay results with emphasis on type of amino acid and/or reaction condition. Yet, as any amine groups attached directly to the ns-nHA surface are not free to react until the ns-nHA is digested, the difference between the two protocols still gives some indication of the relative amount of amine groups involved in such an interaction. Altogether, the data suggests that the attachment of aspartic acid to the ns-nHA was more balanced and sparse than the other two amino acids studied.

## Discussion

Crystalline HA is hexagonally close packed with its unit cell consisting of Ca^2+^, PO_4_^3−^ and OH^−^ groups closely packed together in a hexagonal arrangement^19,22^. While OH serves as the backbone, the 6 phosphates helically arranged around the c-axis are responsible for being the skeletal frame that stabilizes HA^22^. With a Ca/P ratio lower than the stoichiometric value for HA (i.e. 1.67) there are calcium vacancies on the surface of our ns-nHA. Assuming the substitution of tetrahedral phosphate with a planar carboxylate ion during the fabrication of nHA, the negative charge will have been reduced (i.e. increased zeta potential) and fewer calcium ions then necessary to balance the charge. As a result, the surface calcium atoms are likely covered by excess phosphate ions which, when protonated during pre-conditioning in distilled water, will produce P-OH groups^9,23,24^. Particles of high surface area, such as found with nHA, are more likely to exhibit this thermodynamically stable surface deficiency in calcium, as a result of a dissolution and re-precipitation mechanism^25^. An additional characteristic of the as-received, unmodified ns-nHA is its low crystallinity and, as a result, some heterogeneity at the surface is expected. However, bone itself has a mineral phase crystallinity of 51–59%^26^ and as a result of our interests in utilizing the surface-modified nHA in bone-mimicking composites, ns-nHA is found to be acceptable in the investigation presented here.

Blank ns-nHA (i.e. unfunctionalized) controls under regular experimental conditions (i.e. pH 7.8 and unsaturated) are negatively charged as a result of calcium vacancies and the acidic pK_a_ of the ns-nHA surface^2,9^. By pre-saturating the blank ns-nHA with calcium, the zeta potential becomes less negative, while increasing pH also makes this value less negative (although to a lesser degree than calcium saturation). The latter observation may be explained by the relative instability of the ns-nHA surface while held at pH 9 and potential loss of phosphate groups (and/or increased calcium ion retention). The zeta potential dependence on the type of amino acid at the ns-nHA surface may be explained by a “cone” of adsorbance^27^ and greater fraction of unbound calcium upon carboxyl group attachment (for aspartic acid), compared to amine group attachment (for glycine and lysine). The lack of difference between zeta potential values for glycine- and lysine-functionalized ns-nHA may be explained by a slightly more disordered layer attachment of these two amino acids (compared to aspartic acid). Meanwhile, the impact of calcium saturation on zeta potential can be explained by increased repulsion between divalent calcium on the ns-nHA surface and amine groups of the amino acids, particularly for lysine which has a net positive charge. Overall, the change in zeta potential observed upon amino acid absorbance on ns-nHA surface is a result of the complex balance of phosphate ion loss, differences in calcium ion retention, and the additional charge associated with amino acid terminus and side groups. Furthermore, the degree to which the carboxyl and amine functional groups are ionized at a given pH will also impact attachment and resultant zeta potential of the functionalized particles. For example, while carboxyl groups are essentially all negatively charged at both pH conditions studied, less than 80% of the amine groups on aspartic acid and glycine are positively charged at pH 9. Meanwhile, for lysine the alpha-amine group (pK_a_ of 8.95) is less than 50% ionized at pH 9. The fact that pH alone was not found to have a significant impact on zeta potential indicates the likely impact of not only heterogeneity at the ns-nHA surface prior to modification, but also the multi-modal attachment of amino acids to the surface (i.e. not solely governed by either the charged carboxyl or amine groups).

The significant increase in the normalized COO^−^ peak area for blank ns-nHA upon increasing pH can be explained by the degradation of ns-nHA and loss of the phosphate anion, particularly in the absence of amino acids. The ATR-FTIR peak area analysis suggests that the factor of greatest impact on amino acid functionalization of ns-nHA in this study was the type of amino acid itself. In addition, the greater normalized COO^−^ and NH_2_-H^+^ peak areas for glycine and lysine functionalized ns-nHA compared to that for aspartic acid (particularly once accounting for number of functional groups per mole of amino acid) is related to the relative fraction of available sites grafted. A limitation for this study results from the ATR-FTIR peak fitting analysis and the normalization of the data to the PO_4_^3−^ vibration peak. Owing to hydrolysis of the ns-nHA powder during the experiment, the amount of phosphate species present after the reaction likely differ between sample groups (with the presence of amino acids and buffer solution impacting the hydrolysis reaction). As a result, the normalized peak areas of COO^−^ and NH_2_-H^+^ of amino acid functionalized ns-nHA match the general trend of the ninhydrin and fluoraldehyde protocols, but not the difference in magnitude between sample groups. To overcome this limitation, the latter protocols are needed to give a more complete analysis of the success of our functionalization approach.

**Figure 7:**
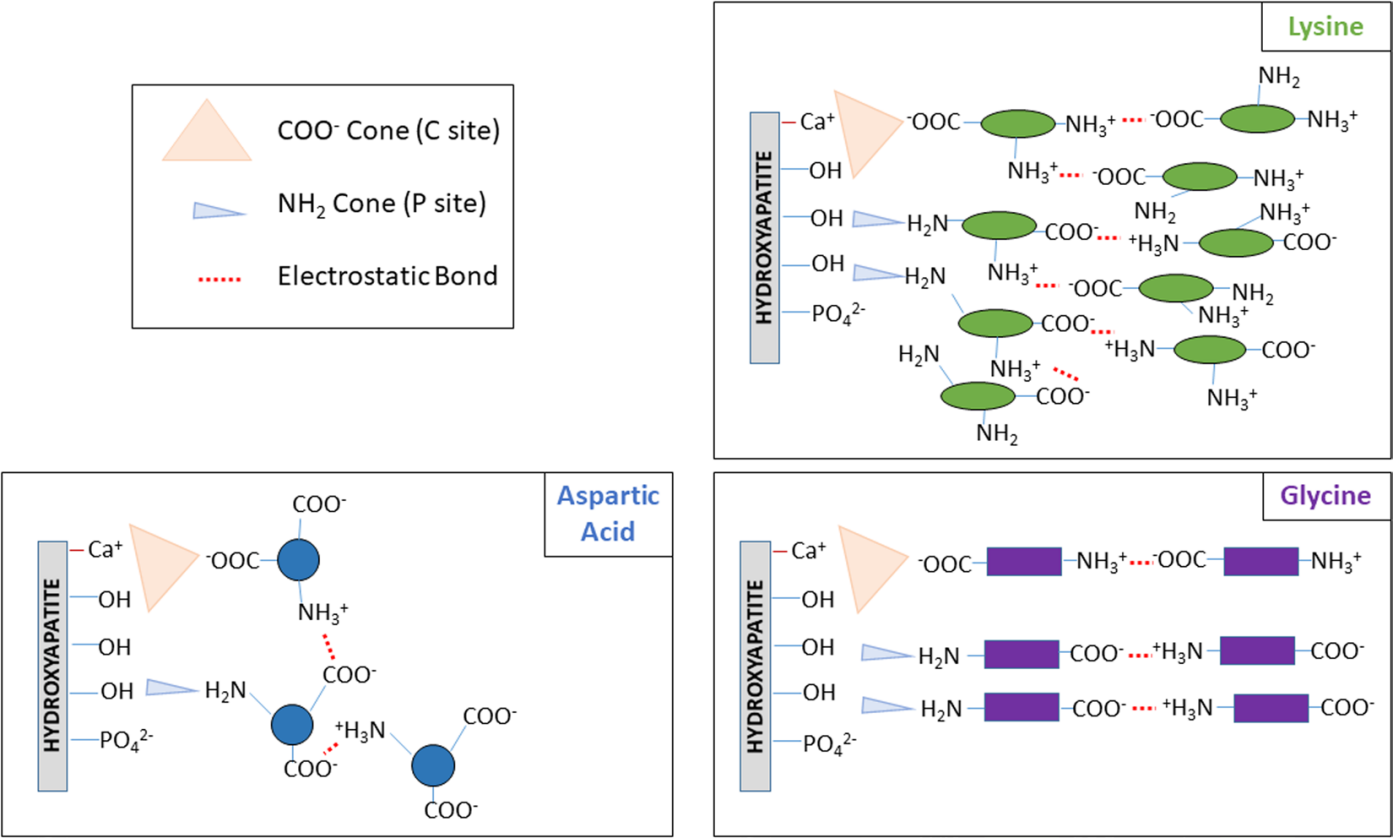
A proposed schematic for the dominant mode of adsorption of the different amino acids at the ns-nHA surface. Both amine and carboxyl functional groups contributed to the attachment of the amino acids to the ns-nHA surface. This schematic generally encompasses each reaction condition, owing to little dependence observed on reaction condition (i.e. pH or pre-saturation with calcium); however, it is important to recognize that the fraction of amine groups which are ionized will change in the schematic as pH changes. Overall, the ease of approach towards the negatively charged ns-nHA surface is greatest for Lysine and Glycine, and more challenging for the negatively charged Aspartic acid. As a result, the attachment of Aspartic acid to the ns-nHA surface is more limited compared to the other two amino acids.

On the surface of ns-nHA, there are two main binding sites (termed C and P sites)^18,28^. The C site is a calcium-rich region which binds strongly to anionic groups such as COO^−^, while the P site is phosphate-rich and interacts with cationic groups such as NH_3_^+^. From our studies, we conclude that each amino acid predominantly binds to the P sites of our negatively charged ns-nHA as a result of strong hydrogen bonding; however, C sites also have a detectable and meaningful amount of binding via electrostatic interactions. With “water-reacted” nHA^11^ there will be a considerable number of POH functional groups (at the P sites); these will form particularly strong hydrogen bonds with the amine groups of the amino acids. Figure 7 provides a schematic for the dominant mode of interaction between the ns-nHA surface and the different amino acids.

The quantified coverage of available ns-nHA graft sites suggests an increased ease of approach of the more positively charged amino acid (i.e. lysine) toward the negatively charged ns-nHA surface under each reaction condition compared to the negatively charged amino acid (i.e. aspartic acid). Aspartic acid attachment was more limited partly due to both it and ns-nHA having net negative charges and some electrostatic repulsion likely in the bulk medium. In addition, the number of graft sites truly available for functionalization should also be discussed. For example, prior work by Garcia-Ramos *et al*.^27^ theorized that the effective volume of adsorption is best modelled by a cone, where any cation located within this cone projection on the surface could not function as an adsorption site. As the cross-sectional area of a COO^−^ group has previously been reported by Shafei *et al*.^23^ to be as high as 20.5 A^2^ and that of NH_3_ found to be 7.0 A^2^, the cone projection is greater for amino acid attachment via the carboxyl groups. As a result of this ‘cone’, the number of sites truly available for aspartic acid attachment via carboxyl groups is less than would be theoretically predicted based on amine attachment. The type of amino acid may also impact the size of these respective cone projections^9^. In addition, the model used to calculate the theoretically expected number of grafting sites is based on a fully crystalline material^20^. As our ns-nHA is not fully crystalline, it is important to recognize that the ninhydrin and fluoraldehyde assays in effect present a scaled comparison of the success of different amino acid attachment to the surface of ns-nHA. The use of two different protocols to present a pre- and post-digestion comparison is another limitation of this study. However, the difference between the two protocols still gives some indication of the relative amount of amine groups involved in such an interaction. Meanwhile, it is interesting to note that the pre-saturation of ns-nHA with calcium does not greatly improve the attachment of any amino acid. This may be explained by a very small reduction in the negativity of the ns-nHA surface upon pre-saturation (as observed by zeta potential measurements). As a result, pre-saturation with calcium largely did not impact the ease with which amino acids may bind to the negatively charged ns-nHA via negatively charged carboxyl groups. Furthermore, as the fraction of total sites grafted in accordance with the post-digestion fluoraldehyde assay is more than 100% for each of the amino acids studied, it is likely that these amino acids attach in relatively thick layers at the ns-nHA surface. While prior investigations have reported that the affinity constants for amino acids are relatively weak^2^, our work suggests that the interaction between amino acid and the ns-nHA surface is notably mediated by the side group. With the molar ratio of amino acid to available ns-nHA sites for grafting being detectably lower than either glycine or lysine, the functionalization control is likely greatest with aspartic acid. In building a bone-mimicking composite, the use of aspartic acid for a more controlled and relatively stable layer functionalization of ns-nHA surface may be beneficial. Future investigation will consider reducing the ratio of amino acid to the ns-nHA in solution in order to improve the efficiency in the reaction and further limit the thickness of an amino acid layer on the ns-nHA surface.

## Conclusion

In this study, we investigated the binding of three different amino acids for the surface of poorly crystalline, non-stoichiometric carbonated nHA (ns-nHA) under different experimental pH conditions and following pre-saturation of ns-nHA with calcium. The attachment affinity at the ns-nHA surface was found to be significantly greater for an amino acid with a basic (i.e. lysine) or non-polar side group (i.e. glycine) compared to an acidic side group (i.e. aspartic acid). This side group dependence was also influential on mode of amino acid attachment to the surface of ns-nHA. For example, both the amine and carboxyl groups significantly contributed to the attachment of the amino acids to ns-nHA; however, the relative proportion of amine group attachment differed depending on the amino acid. Furthermore, as the molar fraction of amino acids bound was greater than 1 following the fluoraldehyde assay, there appears to be a relatively thick layer of amino acid attaching on the ns-nHA surface. By manipulating the experimental conditions for ns-nHA surface functionalization, this study has notably improved the current understanding of amino acid affinity for the surface of nHA.

## Methods

### Characterization of As-Received ns-nHA Powder

Hydroxyapatite nanoparticles were purchased from MKNano (division of M K Impex Corp, Canada). To confirm the shape and size of the starting powder, scanning electron microscopy (SEM; Zeiss Merlin FESEM 1530) was performed, while SEM-energy dispersive x-ray analysis (SEM-EDX) was used to confirm powder composition. Prior to SEM analysis, powders were sputter coated in a magnetron sputtering machine for 120 s to a form an Au film on the particles. In addition, a 4-circle high-resolution x-ray diffractometer (XRD; PANalytical Xpert Pro MRD) was used to confirm ns-nHA crystallinity, and BET (Brunauer, Emmett and Teller) analysis was used to characterize the surface area of the powder.

### Amino Acid-Functionalization of ns-nHA Powder

**Figure 8:**
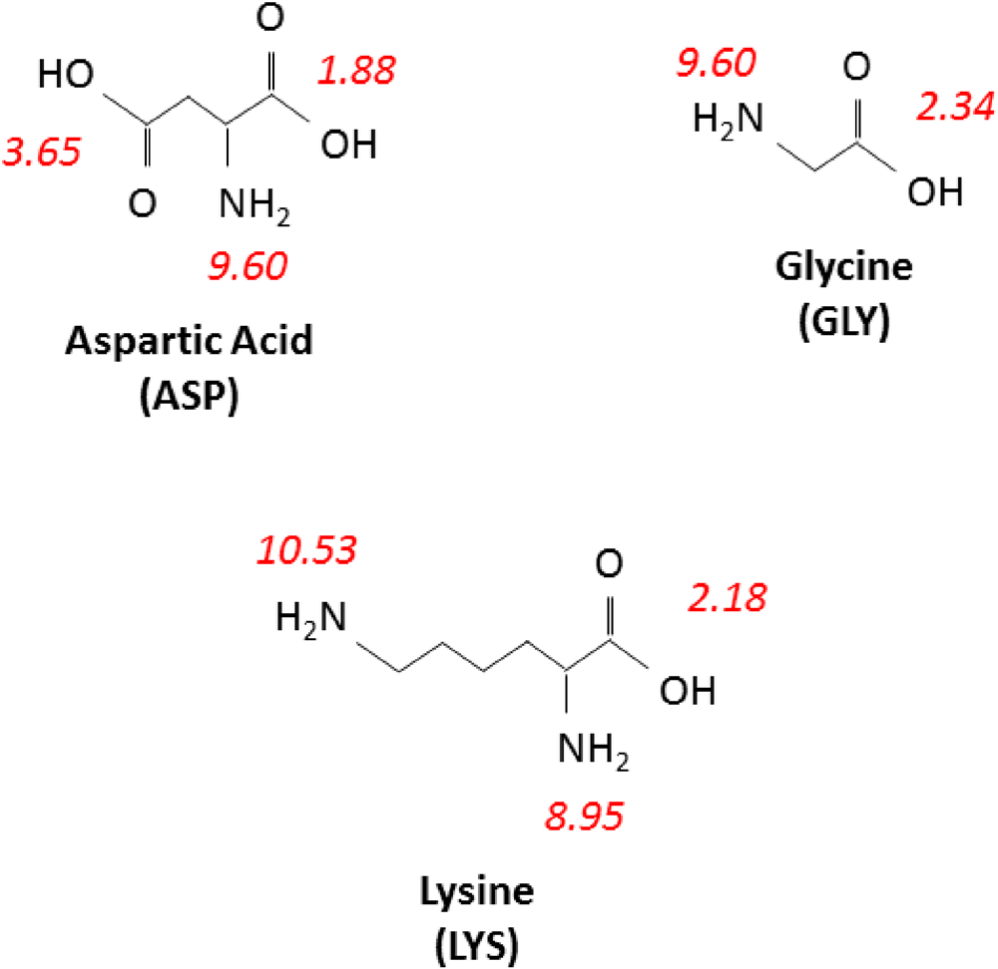
Molecular structure of Aspartic acid, Glycine, and Lysine with pK_a_ values of amine and carboxyl groups represented near corresponding functional groups (pK_a_ values from Nelson *et al*.^31^).

The amino acid reagents used in this study were L-aspartic acid, glycine, and L-lysine mono-hydrochloride (all purchased from Sigma-Aldrich Canada Co). The three amino acids were selected based on the differences in their side chains and net charges under reaction conditions. For example, the pI of glycine is 5.97, of lysine is 9.74, and of aspartic acid is 2.77^2^. Figure 8 depicts the molecular structure and pK_a_ of the amine and carboxyl groups for these three amino acids.

The side chain group of glycine (-H) is non-polar, while that of aspartic acid (-CH_2_COO^−^) is acidic and of lysine (-CH_2_CH_2_CH_2_CH_2_NH_3_^+^) is basic. As a result, at a reaction pH of 7.8, lysine has a net positive charge, the aspartic acid has a net negative, and glycine is slightly negative. At a reaction pH of 9.0, the net charges remain similar in sign, but slightly more negative in magnitude in each case. Stock solutions of glycine and lysine at a concentration of 0.425 M were prepared by adding the requisite mass of powder in 500 mL distilled water (dH_2_O). Meanwhile, as aspartic acid is minimally soluble in dH_2_O^29^, its 0.425 M stock solution was prepared by adding the requisite amount of powder to 200 mL of 2.0 M sodium hydroxide solution (NaOH_(aq)_) until dissolved, and then toping the volume to 500 mL with dH_2_O. The amino acid solutions were stored at room temperature until use.

To ensure the saturation of any calcium deficiencies on the hydroxyapatite surface and decrease the net negativity of the surface charge, 12 g of ns-nHA powder was added to 300 mL of 5.0 mM CaCO_3_ solution and placed on a horizontal shaker at 200 rpm for 3 days. This ns-nHA powder was subsequently termed “CaS” (or calcium saturated). In order to match these pre-conditioning steps without the addition of further calcium, ns-nHA was conditioned under regular unsaturated conditions (labelled “US”) prior to amino acid addition. Under these regular conditions, 12 g of ns-nHA powder was added to 300 mL of dH_2_O on a horizontal shaker at 200 rpm for 3 days. After 3 days of pre-conditioning the pH of the ns-nHA suspensions was recorded using a Accumet® AB150 pH meter (pH_initial_).

Amino acid solutions were then added to each respective container of ns-nHA-based suspension until a total volume of 600 mL was reached. The pH of the amino acid-ns-nHA suspension was maintained at either 7.8 (the previously determined ‘stable’ ns-nHA aqueous suspension pH) or 9.0 using 1.0 M NaOH_(aq)_ and 1.0 M HNO_3(aq)_, during initial amino acid addition and for an additional 15 min. The container was then returned to the horizontal shaker at 200 rpm for another 3 days. Following amino acid reaction with ns-nHA, the pH of the suspension was recorded using an Accumet^TM^ AB150 pH meter (pH_final_), before the suspension was added to 50 mL conical tubes and centrifuged at 1200 rpm for 5 min. The powder was then collected following removal of the supernatant, and rinsed with 5 mL of fresh dH_2_O before being re-spun at 1200 rpm for another 5 min. The powder was collected after removing this last supernatant and then dried in a 37 °C oven. The final dried powders were stored in glass scintillation vials at room temperature until needed for characterization. The change in pH as a result of the amino acid functionalization reaction was calculated as ∆pH = pH_initial_ − pH_final_. A positive ∆pH indicates a decrease in pH during the 3 days of reaction.

### Zeta Potential Characterization of Amino Acid-Functionalized ns-nHA

Following drying the surface charge and relative suspension stability of the amino acid-functionalized ns-nHA was assessed by characterizing the zeta potential of ns-nHA-dH_2_O solutions with a zeta potential analyzer (Wallis^TM^, Cordouan Technologies) and ZetaQ V1.7.0 software (n = 5). Sample suspensions of 0.5 mg/mL ns-nHA were prepared in MilliQ dH_2_O^30^ and buffered to pH 9.0 using 0.1 M NaOH or 0.1 M HNO_3_ were prepared prior to measurement. A pH of 9.0 was chosen following the approach by Gonzalez-McQuire *et al*.^1^ for zeta potential measurement of HA.

### Specific Surface Area (SSA) Characterization of Amino Acid-Functionalized ns-nHA

Surface area measurements were performed on a Quantachrome Autosorb-iQ/MP using liquid nitrogen as the adsorbent gas. Samples were degassed at 250 °C for 1.5 hours under nitrogen gas and backfilled with helium. BET surface area was determined by using a multi-point BET analysis using 12 adsorption points equally spaced from 0.025 P/P0 to 0.3 P/P0.

### ATR-FTIR Analysis of Amino Acid-Functionalized ns-nHA

The ns-nHA powder was also analyzed by attenuated total reflectance Fourier transform infrared (ATR-FTIR) spectroscopy (Tensor 27, Bruker, Germany) to confirm the adsorption of the three amino acids (n = 3). From the ATR-FTIR spectra, vibrations corresponding to PO_4_^3−^ were identified at 1028, 605, and 565 cm^−1^, while bands indicating COO^−^ and –NH_2_-H^+^ symmetric/asymmetric stretches were identified at 1400–1430/1560–1600 cm^−1^ and 1550–1485/1590–1660 cm^−1^, respectively^1^. The peak area for each of the aforementioned functional groups was calculated using DMFit 2010 Software. The peak areas for the COO^−^ and –NH_2_-H^+^ stretches were subsequently normalized to that of the phosphate vibration at 605 cm^−1^ followed by subtracting the matching peak for the blank ns-nHA in order to give an estimation of the relative amounts of amino acid adsorbed on the ns-nHA.

### Pre- and Post- ns-nHA Digestion Assays for Amino Acid Attachment to ns-nHA

The success of amino acid adsorption was further quantified relative to the theoretical availability of grafting sites using both a pre-digestion ninhydrin colourimetric method and a post-digestion fluoraldehyde method. Firstly, the ninhydrin method involves the reaction of free amine groups with a ninhydrin-ethanol solution, and the formation of a Ruhemann’s purple dye anion under basic conditions which can then be photometrically quantified^20^. Utilizing a modified version of an existing ninhydrin staining protocol by Poli *et al*.^20^, reference amine solutions at a concentration of 1.01 × 10^−3^ M were prepared using the original amino acid stock solutions and absolute ethanol, while a ninhydrin solution at 1.97 × 10^−3^ M in absolute ethanol was prepared. Calibration standard solutions were prepared by mixing 0.5 to 5.0 mL of the reference amine solution with 1 mL of the ninhydrin solution, and topping up the total volume of this standard to 6 mL with absolute ethanol. To 50 mg of amino acid-functionalized ns-nHA powder in a 15 mL conical tube, 1 mL of ninhydrin solution and 5 mL of absolute ethanol were added. The tubes containing either control or sample were added to a hot water bath at 90 °C for 90 min. After 90 min, the tubes were cooled to room temperature and centrifuged at 4000 rpm for 10 min. The supernatant from these tubes was then collected and the remaining powder dried overnight at 37 °C. 150 µL of the supernatant was then added to 96-well plates in triplicate and the absorbance read at 586 nm using a SpectramaxPlus (Molecular Devices) and SoftMax Pro V5.4.1 software (n = 5).

While the ninhydrin method allows for the detection of free amine groups of amino acids otherwise attached to the surface of ns-nHA, any amine groups unavailable (e.g. directly attached to ns-nHA) are not detected. As we suspect that carboxyl groups are not the only significant functional group involved in attaching amino acids to the ns-nHA and the ninhydrin protocol is more efficient at slightly basic conditions, another method is needed to free amino acids from ns-nHA and subsequently quantify their presence. To address this 10 mg of ns-nHA powder was digested by adding 400 µL of 4 N hydrochloric acid and vortexing for 30 s. To this clear solution 2 mL of MQ dH_2_O and 225 µL of 4 N NaOH were added to further dilute and increase the pH to ~5, respectively. Standard solutions were prepared in a range of concentrations for each amino acid studied (4.25 × 10^−5^ to 4.25 × 10^−2^ M). To a black Corning™ 96-well plate 100 µL of sample or control solution was added, with 150 µL of Phthaldialdehyde reagent (OPA; Sigma-Aldrich, Canada) then added to each well immediately prior to fluorescence detection at 455 nm using an excitation wavelength of 340 nm with a plate reader (BioTek® Instruments) (n = 5).

The theoretical calculation for the amount of sites available for grafting was derived using Eq. (1)^20^.

1 $${{\rm{n}}}_{{\rm{sites}}}={\rm{SSA}}\ast [{\rm{8}}/({\rm{6}}\ast {\rm{A}}\ast {{\rm{N}}}_{{\rm{a}}})]$$

where SSA is the specific surface area of the ceramic (m^2^/g), A is the total area of the unit cell (m^2^), and N_a_ is Avogadro’s number. Using the determined concentration of amino acid present on the ns-nHA (n_actual_) surface and this theoretical number of available sites for grafting (n_sites_), the percentage of sites successfully grafted was calculated as [n_actual_/n_sites_] × 100%. It is important to note that this Eq. (1) assumes perfect crystallinity with a regularly repeating unit cell, which is a significant assumption given the low crystallinity of our ns-nHA.

### Statistical Analysis

Differences in the means of study outcomes were analyzed using IBM® SPSS® Statistics software and a two-way ANOVA or three-factor univariate general linear model with a significance value of p = 0.05. In addition, a post-hoc Tukey analysis was performed upon confirming data normality. All data is presented in this paper as mean +/− one standard deviation.

## Electronic supplementary material

Supplementary Information

## Data Availability

The datasets generated and analyzed during the current study are available from the corresponding author upon reasonable request.
