# Supplementary material for: Impact of Side Chain Polarity on Non-Stoichiometric Nano-Hydroxyapatite Surface Functionalization with Amino Acids
Source: Sci Rep. 2018 Aug 23;8:12700. doi: 10.1038/s41598-018-31058-5 (PMC6107576; doi:10.1038/s41598-018-31058-5)
Supplement: Supplementary file 1 — Supplementary Information [file 41598_2018_31058_MOESM1_ESM.docx]

**Supplementary Information**

**IMPACT OF side chain polarity on Non-stoichiometric nano-hydroxyapatite surface functionalization WITH AMINO ACIDS**

**Patricia Comeau^1^, and Thomas Willett^1,*^**

^1^ Composite Biomaterial Systems Laboratory, Systems Design Engineering Department, University of Waterloo, Waterloo, ON, N2L 3G1, Canada

*[thomas.willett@uwaterloo.ca](mailto:pcomeau@uwaterloo.ca)





**Figure S1:** SEM image of ns-nHA powder.


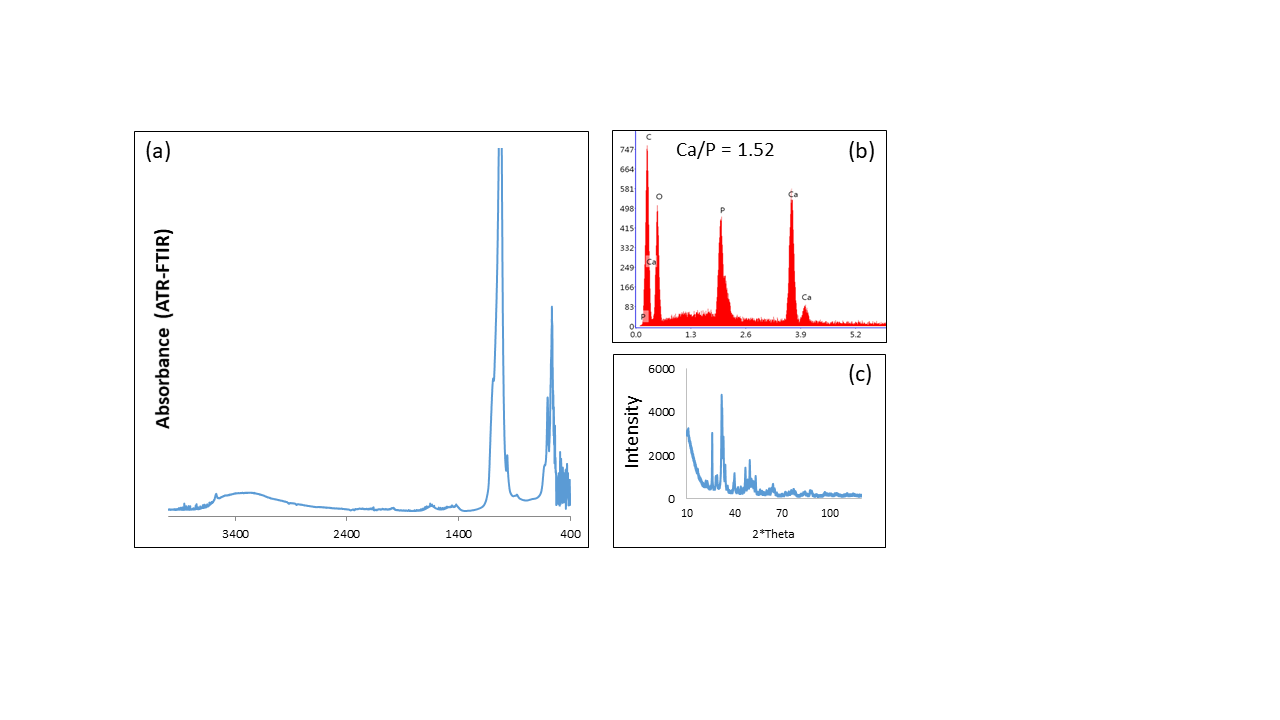


**Figure S2:** Characterization of blank, as-received nHA particles prior to its addition to an aqueous amino acid surface functionalization reaction: (a) ATR-FTIR (b) SEM-EDX and (c) XRD.


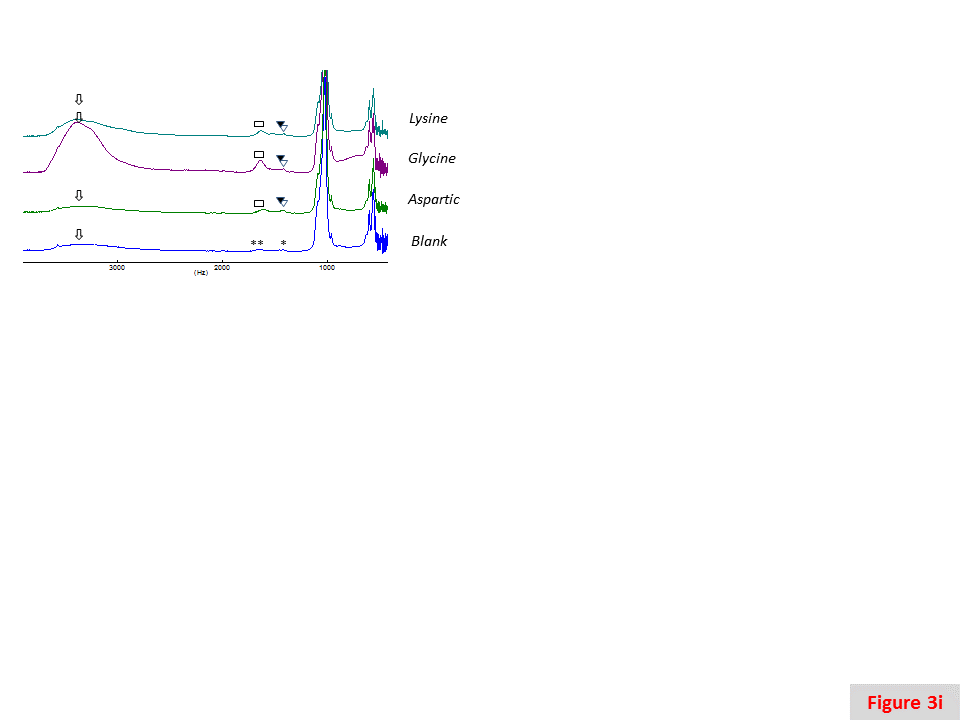


**Figure S3:** ATR-FTIR of Regular, pH 7.8 AA-nHA. Triangles represent symmetric stretching and rectangles represent asymmetric stretching, with hollow shapes for COO^-^ and filled shaped for NH_2_-H^+^. Arrows indicate H_2_O. ** and * indicate the asymmetric and symmetric stretching of COO^-^ in blank (i.e. blank) nHA after 3 days in dH_2_O.


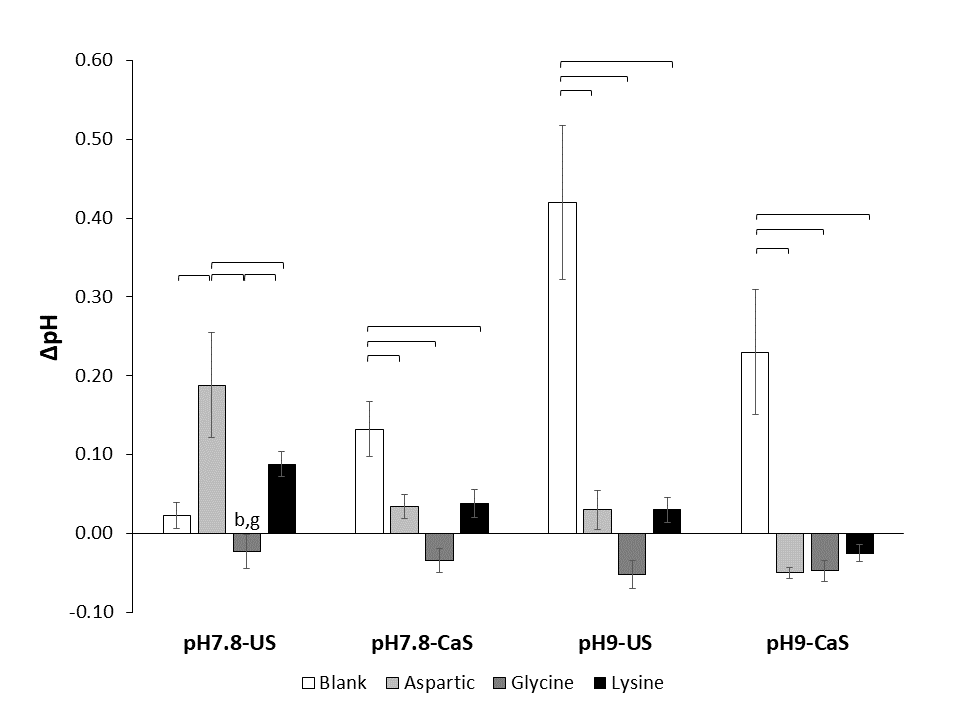


**Figure S4:** pH change during 3-day amino acid surface functionalization reaction of nHA. A positive pH change is indicative of a pH decrease during reaction. Change in reaction pH largely dependent on presence and type of amino acid. Under pH 7.8 and unsaturated (US) conditions the change in solution pH is greater with amino acids present than for the unfunctionalized nHA-containing solutions. Meanwhile, under calcium saturated (CaS) and/or pH 9 conditions the pH change of the unfunctionalized nHA-containing solutions was greater. Data reported as average ± one standard deviation (n=5). Means that do not share a letter have a statistically detectable difference (p<0.05).


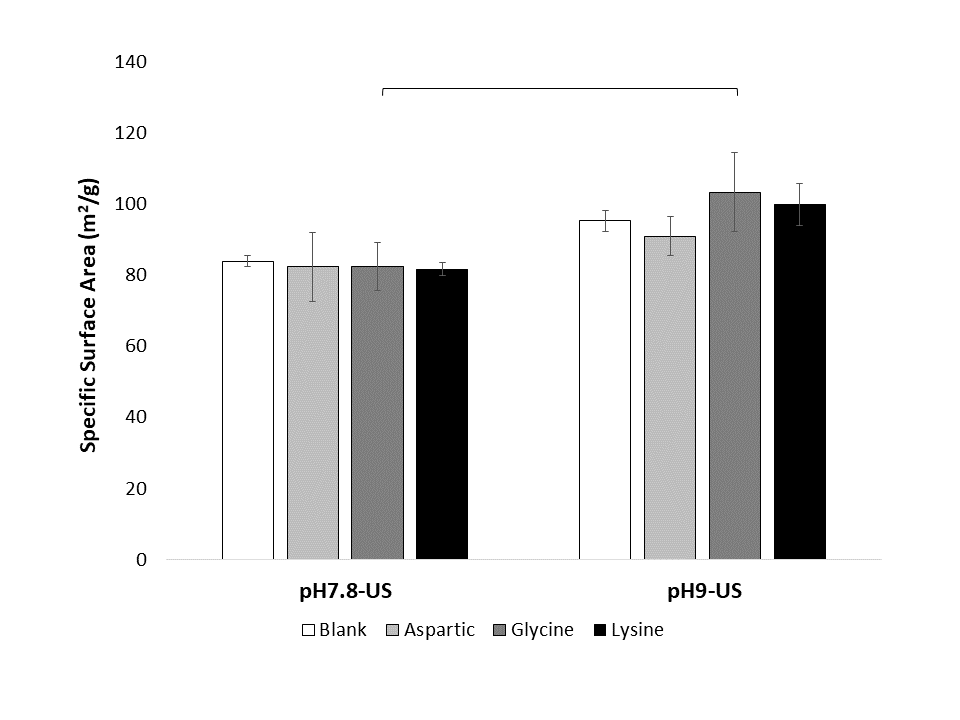


**Figure S5:** Specific Surface Area of functionalized nHA. Reaction pH had a detectably significant impact on specific surface area (p<0.001, two-way ANOVA). Data reported as average ± one standard deviation (n=5). Only nHA unsaturated with Ca^2+^ (given as US) is presented in this figure. Horizontal bars indicate statistically detectable difference (p<0.05).
